# Case control study comparing the HPV genome in patients with oral cavity squamous cell carcinoma to normal patients using metagenomic shotgun sequencing

**DOI:** 10.1038/s41598-021-83197-x

**Published:** 2021-02-16

**Authors:** Ian Ganly, Zhiheng Pei, Yuhan Hao, Yingfei Ma, Matthew Rosenthal, Zhenglin Wu, Jocelyn Migliacci, Bin Huang, Nora Katabi, Wenzhi Tseng, Stuart Brown, Yi-Wei Tang, Liying Yang

**Affiliations:** 1 Head and Neck Service, Department of Surgery, Memorial Sloan Kettering Cancer Center, New York, USA; 2 Department of Pathology, New York University School of Medicine, New York, 10016 USA; 3 Department of Medicine, New York University School of Medicine, New York, 10016 USA; 4 Applied Bioinformatics Laboratories, New York University School of Medicine, New York, 10016 USA; 5 Center for Genomics and Systems Biology, Department of Biology, New York University, New York, NY 10016 USA; 6 Institute of Synthetic Biology, Shenzhen Institutes of Advanced Technology, Chinese Academy of Sciences, Shenzhen, 518000 China; 7 Department of Laboratory Medicine, Memorial Sloan Kettering Cancer Center, New York, USA; 8 Department of Veterans Affairs, New York Harbor Healthcare System, New York, USA; 9 Department of Laboratory Medicine, The Eighth Affiliated Hospital of Sun Yat-Sen University, Shenzhen, China; 10 Department of Laboratory Medicine, The First Affiliated Hospital of Sun Yat-Sen University, Guangzhou, China; 11 Department of Pathology, Memorial Sloan Kettering Cancer Center, New York, USA; 12 Medical Affairs, Cepheid, Danaher Diagnostic Platform, Shanghai, China

**Keywords:** Cancer, Microbiology, Medical research, Pathogenesis, Risk factors

## Abstract

The aim of this study was to carry out a case control study comparing the HPV genome in patients with oral cavity squamous cell carcinoma (OC-SCC) to normal patients using metagenomic shotgun sequencing. We recruited 50 OC-SCC cases which were then matched with a control patient by age, gender, race, smoking status and alcohol status. DNA was extracted from oral wash samples from all patients and whole genome shotgun sequencing performed. The raw sequence data was cleaned, reads aligned with the human genome (GRCH38), nonhuman reads identified and then HPV genotypes identified using HPViewer. In the 50 patients with OC-SCC, the most common subsite was tongue in 26 (52%). All patients were treated with primary resection and neck dissection. All but 2 tumors were negative on p16 immunohistochemistry. There were no statistically significant differences between the cases and controls in terms of gender, age, race/ethnicity, alcohol drinking, and cigarette smoking. There was no statistically significant difference between the cancer samples and control samples in the nonhuman DNA reads (medians 4,228,072 vs. 5,719,715, P value = 0.324). HPV was detected in 5 cases (10%) of OC-SCC (genotypes 10, 16, 98) but only 1 tumor sample (genotype 16) yielded a high number of reads to suggest a role in the etiology of OC-SCC. HPV was detected in 4 control patients (genotypes 16, 22, 76, 200) but all had only 1–2 HPV reads per human genome. Genotypes of HPV are rarely found in patients with oral cancer.

## Introduction

The American Cancer Society estimates about 53,260 people will be diagnosed and 10,750 people will die of oral cancer and oropharyngeal cancer in the United States in 2020^1,2^. More than 95% of these cases will be squamous cell carcinoma (SCC)^1,2^. Although approximately 50% of these patients might be alive 5 years from now, about 20% will die from the disease within 1 year^2^. The 5-year survival of oral cancer has not significantly improved over the past several decades^3^. Although tobacco use is among the strongest risk factors for oral cancer, this factor does not completely explain the incidence of all oral cancer as the drastic decline in the prevalence of cigarette smoking since 1975 (from ~ 40 to 20%) has caused only a moderate change in the incidence of oral cancer^4,5^. This indicates a paradigm shift in the cause of oral cancer and the need to search for other risk factors.

High risk genotypes of Human papilloma virus (HPV), genotypes 16, 18 and 33, now account for 60–80% of all oropharyngeal squamous cell cancers^6–11^. This has led to the hypothesis that some genotypes of HPV may also be responsible for the epidemiology change in the etiology of oral cancer as well. Although the prevalence of the high risk genotypes HPV 16, 18 and 33 varies greatly across multiple studies on oral cancer^12^ it is now accepted that these high risk genotypes are unlikely to be responsible. With regards to other genotypes of HPV, over the past 10 years an increasing number of HPV types have being found in oral samples^13,14^. The currently available HPV detection kits only detect a limited number of HPV genotypes. Since there are over 200 different genotypes of HPV, we hypothesized these may be responsible for some cases of oral cancer. The aim of our study was therefore to carry out a case control study in 50 oral cancer patients and 50 matched control patients to detect all 200 genotypes of HPV using next generation sequencing.

## Methods

To examine the hypothesis that oral HPV is associated with OC-SCC, we performed a case control study with mouthwash samples from 50 patients with OC-SCC and 50 subjects with no oral lesions. Total genomic DNA was extracted from cell pellets of the mouthwash samples. Subject recruitment, sample collection, data generation and analysis are detailed below.

### Recruitment of human subjects for oral cavity squamous cell carcinoma cases and matched controls

A case–control study was approved by the Institutional Review Board of Memorial Sloan Kettering Cancer Center (IRB 15-256) and New York University School of Medicine (i15-00389). All methods were performed in accordance with the relevant guidelines and regulations. Informed consent was obtained from all patients in the study population. From Memorial Sloan Kettering Cancer Center (MSKCC), we recruited 50 oral cavity squamous cell carcinoma (OC-SCC) cases. These cases were then matched with a control patient by age, gender, race, smoking status and alcohol status. Overall, 100 subjects (50 cases and 50 controls) were enrolled in this study. The controls comprised patients with thyroid nodules (benign or malignant). These patients had complete head and neck examination including flexible laryngoscopy and were found to have no evidence or oral cancer. In patients with oral cancer, OC-SCC was confirmed by histological examination of biopsy specimens. Pathological grade and stage of OC-SCC were determined by histopathological examination at the time of surgical resection. Demographic and clinical information was collected for each patient.

### Detection of HPV in OC-SCC tumor samples

High risk (HR) HPV infection was determined by Tissue HR HPV PCR and p16 immunohistochemistry on tumor tissue of OC-SCC patients. Ang et al. has reported that the expression of p16INK4a by immunohistochemistry correlated well (kappa = 0.80; 95% CI 0.73–0.87) with the presence of HPV DNA in tumors^15^. This is cheaper and easier to carry out than ISH and PCR and therefore immunostaining of tumor sections for p16INK4a is now used as an indirect marker for HPV status in clinical pathology laboratories around the world^16^. In prospective randomized trials on treatment of patients with HPV related oropharyngeal cancer, p16 immunohistochemistry is now used as the surrogate marker for HPV positivity in the USA. Rarely some p16 positive tumors may not be HPV related. The addition of HPV PCR to the detection methodology would increase specificity as described by Prigge et al.^17^. In our study, all pathology specimens were examined by a single pathologist specialized in head and neck pathology (NK). p16 immunohistochemistry was performed as follows: four-micrometer tumor sections were deparaffinized, and after heat-induced epitope retrieval, immunohistochemistry for p16INK4a was performed with the primary antibody dilution of 1:7 as per manufacturer’s protocol (CINtec Histology Kit, catalog #9517, Roche mtm Laboratories AG, Heidelberg, Germany). Cases with nuclear and cytoplasmic immunolabeling in at least 70% of the tumor cells were considered positive for p16. In patients with available tissue, HR HPV PCR was done to confirm results of the p16 immunohistochemistry.

### Detection of all 200 genotypes of HPV in mouthwash samples of OC-SCC patients and control patients using metagenomic shotgun sequencing (MSS)

**Figure 1:**
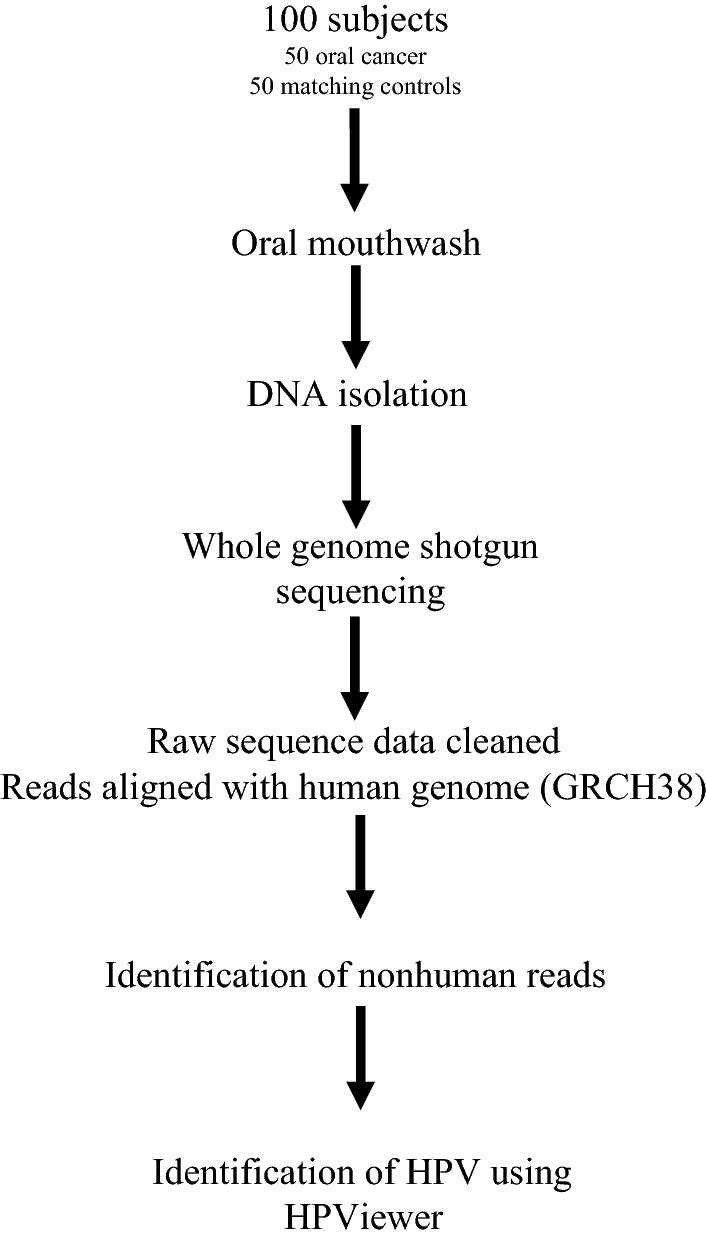
The workflow for the collection and processing of samples and detection of HPV by DNA sequencing.

The detection of HPV in oral rinse samples and saliva samples using PCR has been reported as being a sensitive and specific method for the detection of HPV related oropharyngeal cancer^18–25^. This technique has been reported as a potential screening method for HPV oropharynx cancer^18–22^ and also for the detection of persistent disease or recurrent disease following treatment in patients with HPV related oropharyngeal cancer^23–25^. We therefore used oral rinse specimens from patients with oral cavity cancer and patients with no head and neck cancer for the detection of HPV genotypes. The workflow for the collection and processing of samples and detection of HPV by DNA sequencing is shown in Fig. 1 and detailed as follows:

#### Mouthwash sample collection, processing, storage and DNA extraction

The participants rinsed their mouth vigorously with 10 ml sterile saline for 30 s and then mouthwash collected in a 50 cc falcon canical flask container. After centrifugation at 3120*×g* for 20 min, supernatants were decanted and then the cell pellets were transferred into a 2-ml Eppendorf tube and stored at − 80 °C freezer for further study. All oral rinse specimens were taken prior to surgical resection of the OC-SCC. These samples were de-identified and coded. Using the MoBio method, we successfully extracted DNA from all 100 oral wash samples. DNA yield was measured by the Nanodrop method.

#### Library preparation and samples sequencing

The DNA fragmentation and shotgun metagenomic library construction and sequencing was carried out at the BGI Americas Corp (Cambridge, MA) using Kapa kit and Illumina HISeq X Ten, with 100 samples pooled into 8 lanes.

#### Raw sequence data quality control

The Illumina sequencing process supplied raw sequence reads in fastq format and assigned a quality score called ‘Phred scores’ to describe the base accuracy. The raw sequence quality was reviewed using FASTQC software and any adapters and low-quality reads were removed using Trimmomatic. Low quality reads were defined as leading low quality or N bases, quality < 25; trailing low quality or N bases, quality < 25, scanning the read with a 4-base wide sliding window and cutting when the average quality per base drops below 25 and drops reads below 50 bases long. On average, each sample yielded 70,984,784 ± 7,145,632 raw reads, ranging 54,314,162 to 88,936,078. After trimming, 43,400,676 ± 5,586,559 reads left, ranging 32,127,260 to 58,483,416. The average percentage of clean to raw reads was 61.16% (Minimum: 47.12%; Median: 62.24%; Maximum: 72.69%) (Supplementary Fig. 1).

#### Identification of non-human DNA reads

All trimmed reads were aligned to the human genome (GRCh38). Those with ≥ 90% similarity were considered as human reads. On average, each sample yielded 36,184,844 ± 7,255,847 human reads, ranging 18,446,834 to 56,005,990. The sequencing depth was estimated by averaging the coverage across all 22 human autosomal chromosomes. There was no statistically significant difference between the cancer samples and control samples in the sequencing depth (medians 33,760,382 vs. 34,046,615, P value = 0.77, Mann Whitney test) (Supplementary Fig. 2).

#### Detecting and genotyping HPV in mouthwash samples obtained from patients with OC-SCC and healthy controls

We compared HPV prevalence and abundance between the 50 patients with OC-SCC and 50 subjects with no oral lesions. The nonhuman DNA reads were searched for HPV DNA using HPViewer (Supplementary Fig. 3). We have developed a pipeline to identify HPV reads generated by MSS based on the sequence similarities to the genome sequences of HPV prototypes and used it in a survey of HPV in healthy subjects^26^. Since then, we have made several improvements to allow more accurate detection and classification of HPV reads in human samples in a new software program HPViewer^27^. Briefly, we found that HPV shares not only massive amount of homologous sequences among different HPV types but also extensive simple repeats with human and some bacteria. The inter-type homologous sequences cause errors in HPV genotyping and the shared repeats between human and bacteria can be mistaken as HPV DNA. In HPViewer, these shared regions in the reference HPV genomes are masked to minimize these errors. We also replaced BLAST in the old pipeline with Bowtie2, an ultrafast and memory-efficient tool for aligning sequencing reads to long reference sequences. We did not have a positive control group in the study. However, in the study to develop the software program HPViewer^27^, we evaluated the metagenomic methods and HPViewer with six tumor tissue specimens and matched oral washes from patients with recurrent respiratory papillomatosis (RRP). HPViewer detected HPV reads in all tumor samples [288–4229 HPV (type 6 or 11) reads per tumor sample] while in the six matched oral wash samples, only two were positive for the concordant HPV types with only 2 HPV reads/sample. Because the 2 HPV reads in the oral wash samples shared the same SNPs with the HPV reads in the corresponding tumor samples, it was concluded that the oral HPV reads represent the HPV released from the tumors in the larynx. In various oral cavity sites in healthy people, HPV prevalence ranged 2.9 to 7.1% and type 16 occasionally detected^27^. Thus, the low level HPV reads detected in the oral washes samples most likely represent HPV released from the tumors or from infection site in the oral cavity.

## Results

### Patient characteristics of cases and controls

Patient demographics are shown in Table 1. There were no statistically significant differences between the cases and controls in terms of gender, age, race/ethnicity, alcohol drinking, and cigarette smoking. In the 50 patients with OC-SCC, the subsite was tongue in 26 (52%), floor of mouth in 8 (16%), lower gum in 7 (14%), upper gum in 4 (8%) (Table 2). All patients were treated with primary resection and neck dissection.

**Table 1:** Patient characteristics of cases and controls.

Characteristics	OC-SCC (n = 50)	Normal controls	P value
**Sex (%)**			
Women	23 (46%)	23 (46%)	1^a^
Men	27 (54%)	27 (54%)	
**Age (mean ± SD)**	62.1 ± 11.9	61.6 ± 11.7	0.827^b^
**Race (%)**			
White	44 (88%)	44 (88%)	0.765^a^
Others	6 (12.0%)	6 (12%)	
**Alcohol drinking (%)**			
Never/quit	18 (36%)	17(34%)	0.364^a^
Moderate	27 (54%)	23 (46%)	
Heavy	5 (10%)	10 (20%)	
**Smoking (%)**			
Never	22 (44%)	24 (48%)	0.67032^a^
Quit	20 (40%)	21 (42%)	
Active	8 (16%)	5 (10%)	

^a^Chi-square test.

^b^*T* test, two tailed.

**Table 2:** Tumor characteristics of oral cancer patients.

Characteristic	No (%)
**Tumor subsite**	
Tongue	26 (52)
Floor of mouth	8 (16)
Upper gum	4 (8)
Lower gum	7 (14)
Buccal	2 (4)
Retromolar trigone	2 (4)
Lip	1 (2)
**Tumor size (mm)**	
1–10	11 (22)
11–20	15 (30)
21–30	12 (24)
31–40	8 (16)
41–50	4 (8)
**Pathology T stage**	
T1	24 (48)
T2	14 (28)
T3	4 (8)
T4	8 (16)
**Pathology N stage**	
N0/Nx	29 (58)
N +	21 (42)
**Tumor grade**	
Well differentiated	10 (20)
Moderately differentiated	32 (64)
Poorly differentiated	8 (16)
**Tissue p16 immunostain**	
Positive	2 (4)
Negative	47 (94)
Not done	1 (2)
**Tissue HR HPV PCR**	
Positive	1 (2)
Negative	25 (50)
Not done	24 (48)

### Tumor characteristics of OC-SCC

Pathology details are shown in Table 2. Of the 50 OC-SCC patients, 37 (74%) had pathological T1T2 tumor and 21 (42%) had a pathological positive neck. The majority of primary tumors were either well (20%) or moderately differentiated (64%). All but 3 tumors were negative on p16 immunohistochemistry. Of 26 samples tested by HPV PCR, 25 were negative for high risk HPV and only 1 was positive. The positive HPV PCR case was also positive on p16 imunohistochemistry.

### Metagenomic HPV sequencing results of mouthwash samples of OC-SCC and control patients

The DNA yields ranged 63 ng/µl (range 11.1–127.8) for the cases and 54.3 ng/µl (range 12.6–106.5) for the controls.

#### Detection of nonhuman DNA reads

**Figure 2:**
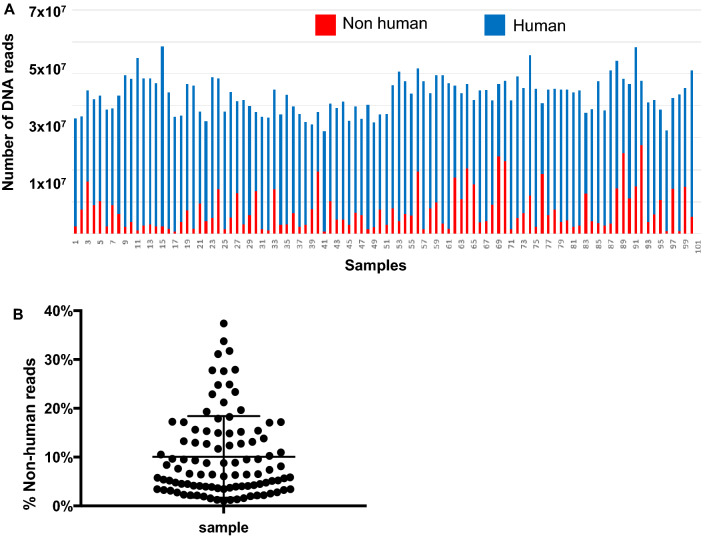
Distribution of nonhuman DNA reads. Number (**A**) and percentage (**B**) of nonhuman reads were calculated along with human reads and total reads. The Y axes show the percentage of nonhuman reads over the total reads. The X axes show sample distribution within the range. Mean reads ± SD is shown in Panel (**B**).

**Figure 3:**
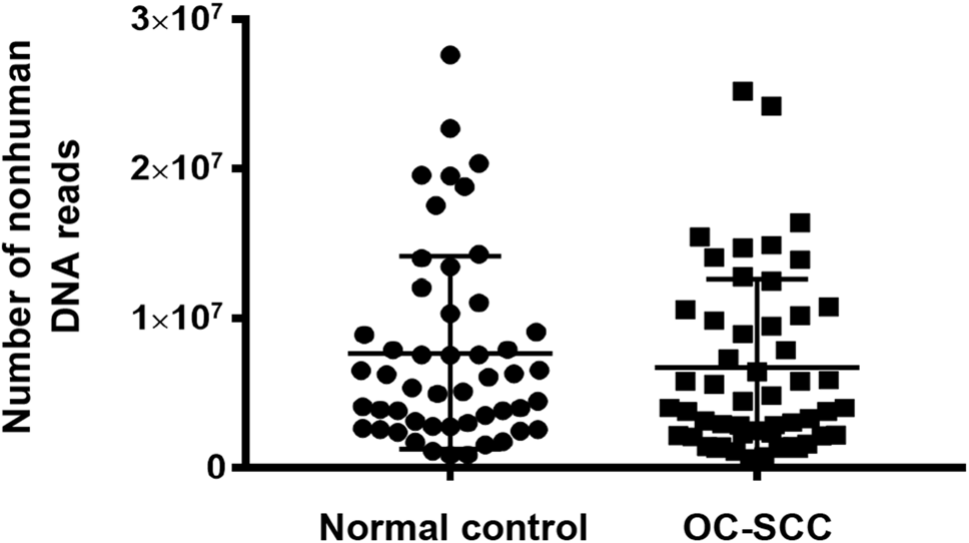
Comparison of OC-SCC and normal controls in nonhuman DNA reads.

Nonhuman reads averaged 7,215,832 ± 6,165,570, ranging 601,022 to 27,624,778. On average, nonhuman reads accounted for 10.06 ± 8.36% of the total reads, ranging 1.01% to 37.37% (Fig. 2). There was no statistically significant difference between the cancer samples and control samples in the nonhuman DNA reads (medians 4,228,072 vs. 5,719,715, P value = 0.324, Mann Whitney test (Fig. 3).

#### Detecting and genotyping HPV in mouthwash samples obtained from patients with OC-SCC and controls

Using HPViewer, HPV was detected in five cases (10%) of OC-SCC and four controls (8%) (Table 3). The raw data for HPV reads is accessible at http://www.ncbi.nlm.nih.gov/bioproject/692713 using the BioProject ID PRJNA692713. In the 5 OC-SCC cases, only 1 tumor **s**ample (sample 90) yielded a considerable number of HPV reads suggesting a role in the etiology of OC-SCC in this patient. This was genotype 16. This patient was also positive on tissue HPV by PCR and positive p16 immunohistochemistry. The location of the tumor was the anterior 2/3rds of the oral tongue. The other 4 tumor cases yielded only about 1–2 HPV reads per human genome. These were in serotypes HPV 10, 16 (2 cases) and 98. All 4 patients were negative on p16 tissue immunohistochemistry and tissue HPV-PCR. Of the 4 control patients, all had only 1–2 HPV reads per human genome. These were in genotypes HPV 16, 22, 76, and 200.

**Table 3:** HPV genotypes detected by MSS in oral cancer and control patients.

Case	Phenotype	Tumor location	Raw HPV counts	Normalized HPV counts^a^	HPV type
T90	OC-SCC	Anterior 2/3 of tongue	42	52.93	16
T98	OC-SCC	Retromolar trigone extending to buccal mucosa and lower gingiva	2	1.34	16
T101	OC-SCC	Buccal mucosa	2	1.4	16
T78	OC-SCC	Lower gingiva	2	1.49	98
G31	OC-SCC	Lateral border of tongue	1	0.63	10
T71	Control	NA	2	1.52	22
T109	Control	NA	2	2.51	200
T116	Control	NA	2	1.39	76
T123	Control	NA	2	1.37	16

^a^Number of HPV reads per human genome.

## Discussion

Over the past 20 years the epidemiology of oral cancer has been changing. Traditionally, oral cancer has been caused by smoking and heavy alcohol consumption^3^. There has been a steady decline in the use of cigarettes and alcohol in the population^4^. Despite this, the incidence of oral cancer has failed to decline. New studies show that there is an increasing number of patients who do not smoke or drink alcohol excessively but still develop oral cancer^5^. These patients tend to be younger with an increased frequency in females. The cause of oral cancer in these patients remains an enigma.

This change in epidemiology has occurred over the same time period as the change in epidemiology of oropharyngeal cancer. In oropharyngeal cancer it is now recognized that the agent causing cancer is high risk genotypes of the Human Papilloma virus, notably HPV 16, 18, and 33^12^.

This has led several studies to be carried out on oral cancer patients to identify high risk HPV genotypes, either in saliva or in tissue tumor specimens. The results of these studies have been highly variable with some studies showing little association whereas others have reported a strong association^12^. However, it is now generally accepted that the high risk genotypes HPV16, 18, and 33 are unlikely to be responsible for the change in epidemiology or oral cancer. There are 200 different genotypes of HPV. The traditional HPV detection kits/methods cover only a limited number of high/low risk (HLR) HPV genotypes^28–32^. Most of these traditional HPV detection methods are PCR-based and detect 14 genotypes (HPV 16, 18, 31, 33, 35, 39, 45, 51, 52, 56, 58, 59, 66, and 68). Since these detection kits do not cover all 200 different genotypes of HPV, it is possible that these detection methods may be failing to identify other genotypes of HPV which may be responsible for causing oral cancer.

The limited ability of current commercial HPV detection kits can be overcome by metagenomic shotgun sequencing (MSS). This is a non-selective approach that, in theory, permits the identification of all HPV sequences. Recently, MSS has been used to detect HPV in some human samples and to identify several novel HPV types^33–38^. In particular, a study that involved condyloma samples shown to be negative for HPV by traditional PCR revealed the ability of MSS to identify many putative HPV sequences^33^. Using MSS, we surveyed HPV distribution in various body sites of 103 healthy human subjects and found that the majority of the 109 HPV types detected could not be detected using the widely used commercial kits and do not belong to the HLR HPV types^26^. Interestingly, the HPV types detected have strong organ tropism and the oral HPV community is different from that of the vagina. These findings raised the possibility that the oral HPV types that are invisible to the traditional detection methods contribute to the etiology of human diseases, such as OC-SCC. The aim of our study was to carry out a case control study in 50 oral cancer patients and 50 matched control patients to detect all 200 genotypes of HPV by MSS.

In our study our cancer patients and control patients were well matched in terms of age, gender, and smoking and alcohol status. We used oral rinse samples from each patient and extracted DNA from cell pellets prior to sequencing. DNA extracted from oral rinse samples has been reported to be a sensitive and specific method for the identification of patients with oropharyngeal cancer^18–22^ as well as the detection of persistent or recurrent cancer in patients who have completed treatment^23–25^. Our sequencing methodology using MSS was also sound with comparable reads in both tumor and control samples. Through our comprehensive detailed sequencing analysis we have shown that only 1 patient had a tumor sample (2%) that could be directly related to HPV. In this case, it was HPV16 which is the main genotype responsible for oropharyngeal cancer. Importantly, no other genotype of HPV was able to be detected at a high copy number indicating that these other genotypes are not responsible for oral cancer in these patients. These observations are highly relevant because they now provide evidence that HPV is rarely associated with oral cancer. There are 2 other studies which support our findings. A study by Bragelman using RNASeq to sequence the tumor mRNA in 7 patients with oral tongue cancer who did not smoke or drink alcohol failed to show any HPV sequences^39^. Another study by Li et al. examined the tumor transcriptomes of 20 patients with oral tongue cancer and failed to identify any HPV viral transcriptome^40^. A recent metanalysis by Sahovaler reported that in oral cavity locations, overall survival was not significantly associated with HPV positivity (hazard ratio [HR], 1.16; 95% CI 0.83–1.61; I2 = 71%)^41^.

There is much interest in identifying factors responsible for oral cancer in these patients. It is possible other viruses such as herpes simplex, herpes zoster, Epstein Barr virus may be responsible though research on these common viruses have not shown any association to date^40^. Even more research is ongoing to identify bacteria which may be responsible. Studies on the oral microbiome have recently been published suggesting specific bacteria may be responsible^19,42,43^. Our own group recently identified that the periodontal pathogens *Fusobacterium, Prevotella, Alloprevotella* were enriched while commensal *Streptococcus* depleted in OC-SCC in nonsmoking patients with premalignant oral cavity lesions as well as oral cancer^19^. Clearly this is an area which requires much research effort to try to provide new insight into this devastating disease.

In conclusion, our study suggests no role in the aetiology of HPV in oral cavity cancer. However, our population of patients is fairly homogenous population (88% white ethnicity) and all from the USA. It is possible that there may be geographic or ethnic differences in the role of HPV across populations and we therefore cannot extrapolate from a single 50 patient study”. Further research in different geographic and ethnic populations is needed. New research is also needed to explore other infectious agents such as bacteria or viruses that may be responsible for oral cancer. Although we saw few HPV reads in our metagenomic data, it is important to analyse the metagenomic sequences for other non human reads from other viruses, bacteria or even fungi. It is possible these other microbes may have an association with OSCC pathogenesis. To carry out such a comprehensive analysis requires complex bioinformatics as well as validation studies. These studies are currently underway.

## Supplementary Information

Supplementary Figures.

Supplementary Table.

## Data Availability

The raw data for HPV reads is accessible at http://www.ncbi.nlm.nih.gov/bioproject/692713 using the BioProject ID PRJNA692713. The accession codes for positive reads for HPV are given in Supplementary Table 1.
