# Supplementary material for: Case control study comparing the HPV genome in patients with oral cavity squamous cell carcinoma to normal patients using metagenomic shotgun sequencing
Source: Sci Rep. 2021 Feb 16;11:3867. doi: 10.1038/s41598-021-83197-x (PMC7886861; doi:10.1038/s41598-021-83197-x)
Supplement: Supplementary file 1 — Supplementary Figures. [file 41598_2021_83197_MOESM1_ESM.pptx]

## Slide 1
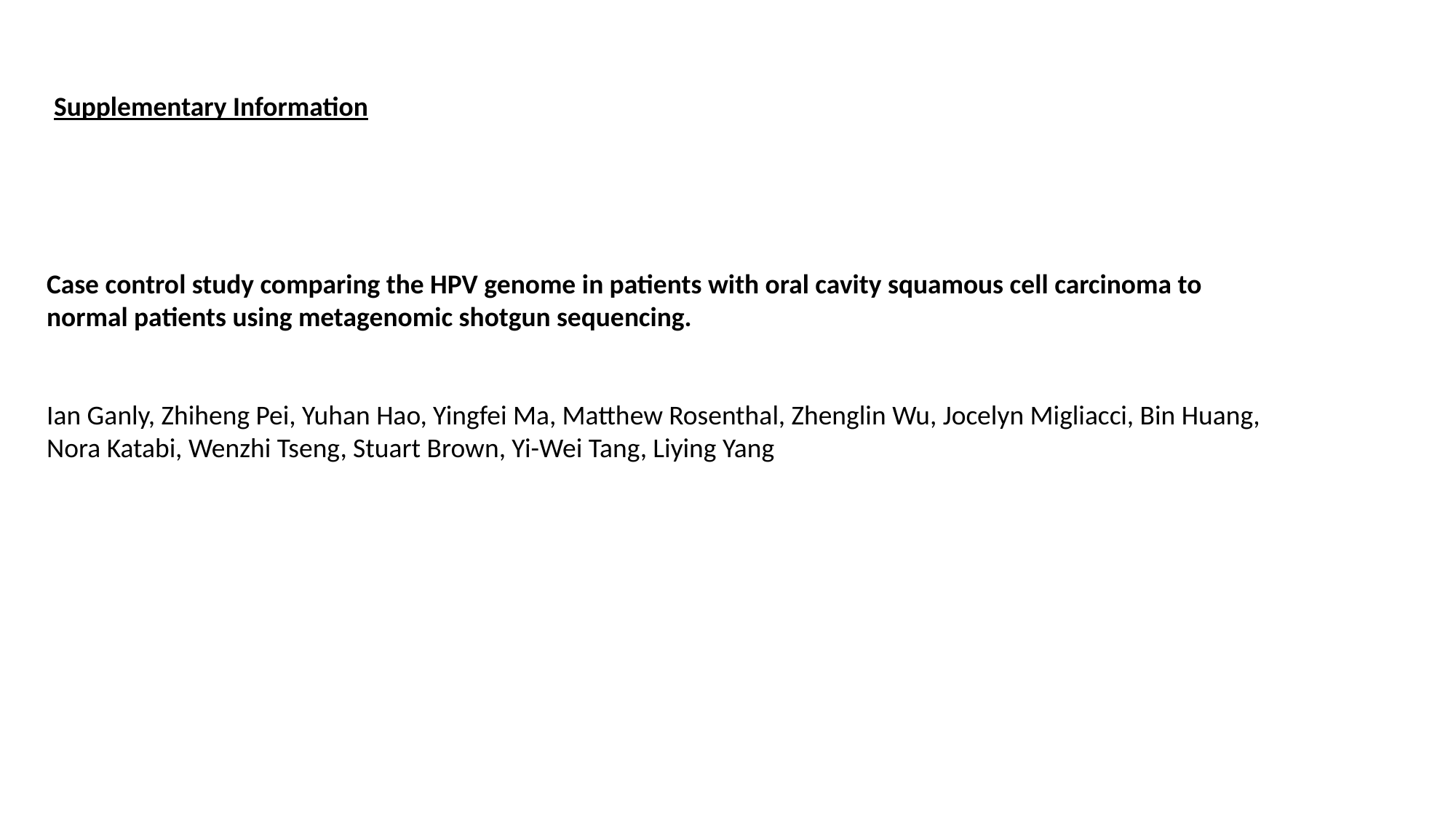

Supplementary Information
Case control study comparing the HPV genome in patients with oral cavity squamous cell carcinoma to normal patients using metagenomic shotgun sequencing.
Ian Ganly, Zhiheng Pei, Yuhan Hao, Yingfei Ma, Matthew Rosenthal, Zhenglin Wu, Jocelyn Migliacci, Bin Huang, Nora Katabi, Wenzhi Tseng, Stuart Brown, Yi-Wei Tang, Liying Yang

## Slide 2
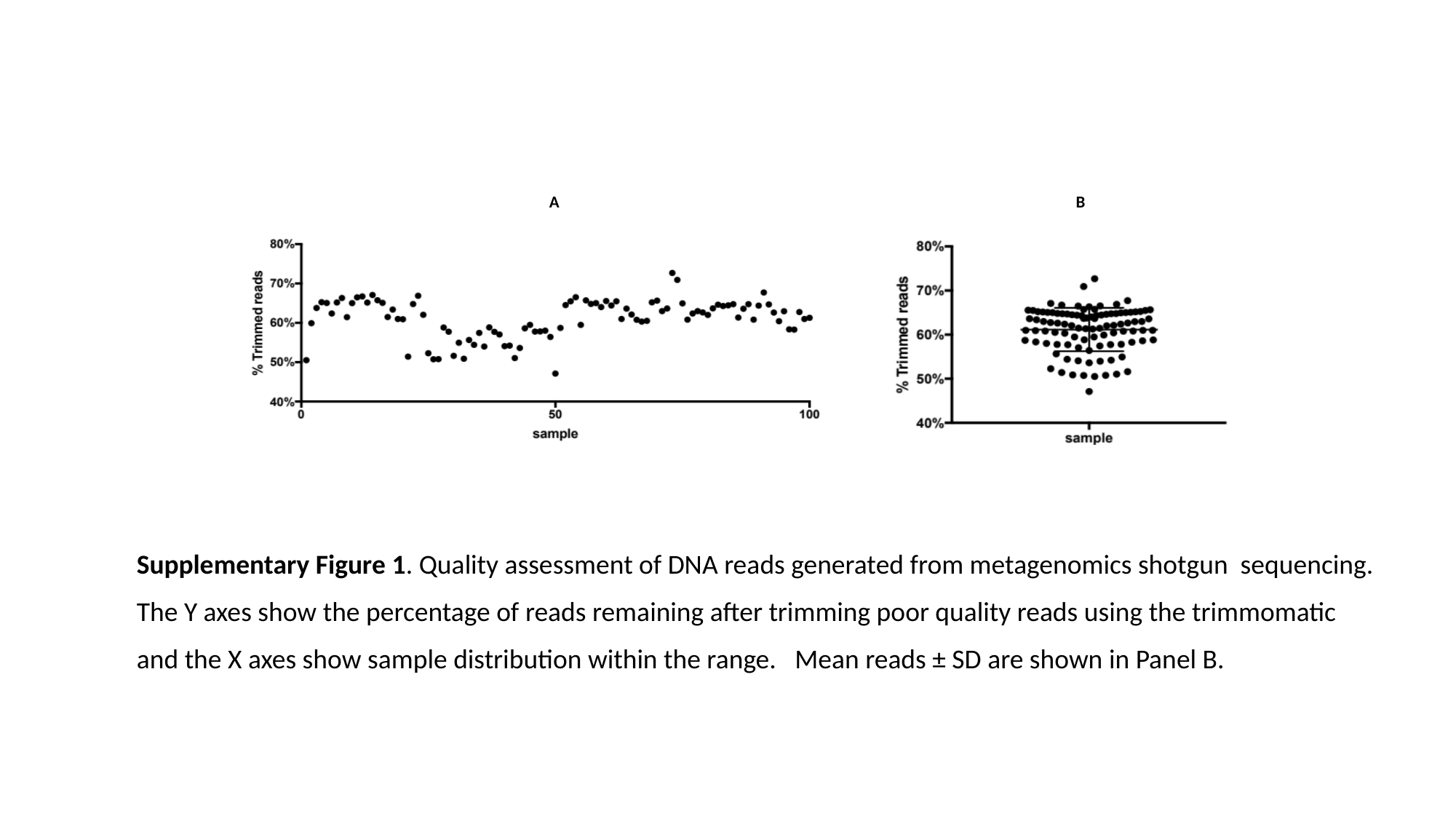

A
B
Supplementary Figure 1. Quality assessment of DNA reads generated from metagenomics shotgun sequencing.
The Y axes show the percentage of reads remaining after trimming poor quality reads using the trimmomatic
and the X axes show sample distribution within the range. Mean reads ± SD are shown in Panel B.

## Slide 3
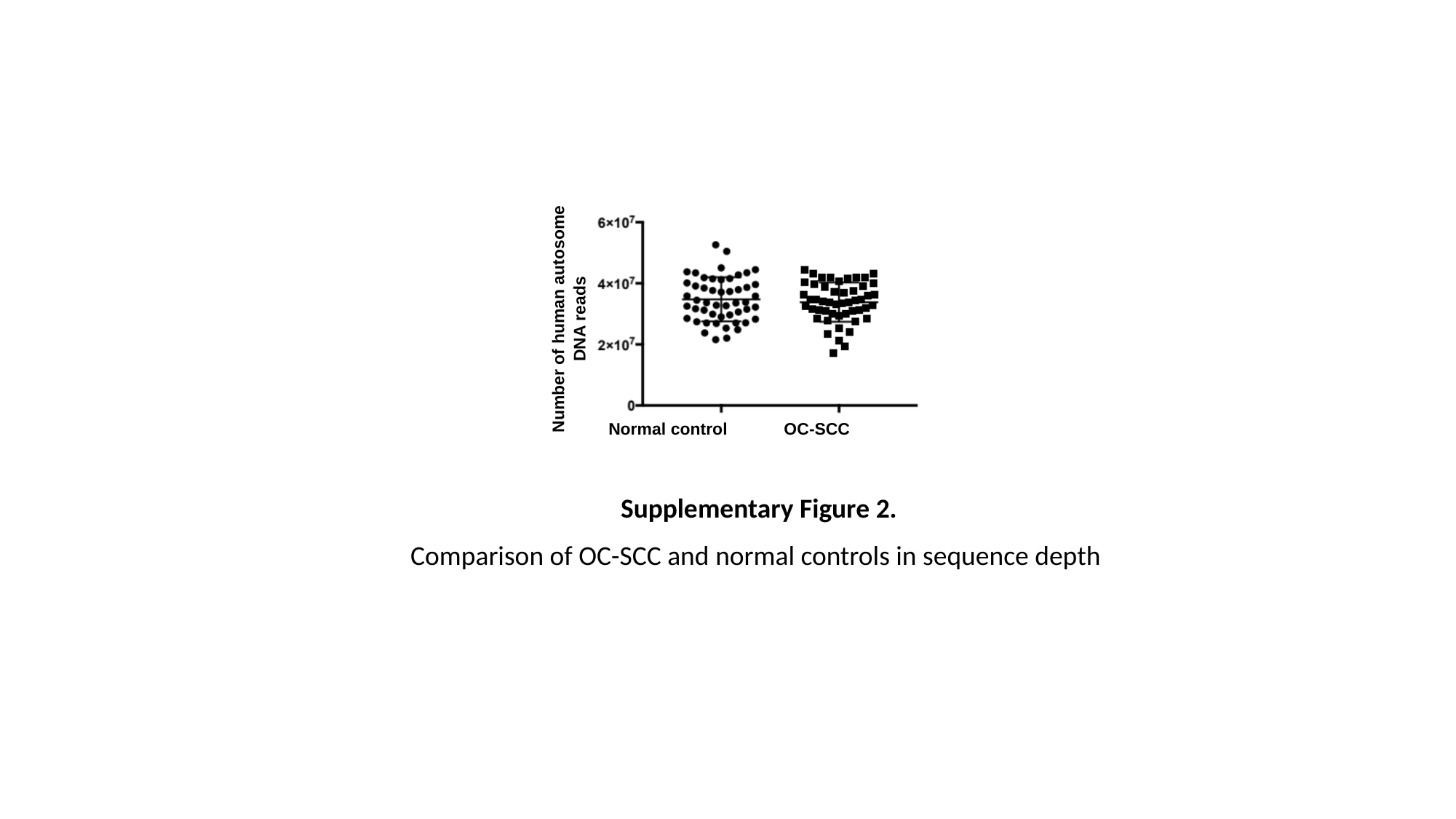

Number of human autosome DNA reads
Normal control OC-SCC
Supplementary Figure 2.
Comparison of OC-SCC and normal controls in sequence depth

## Slide 4
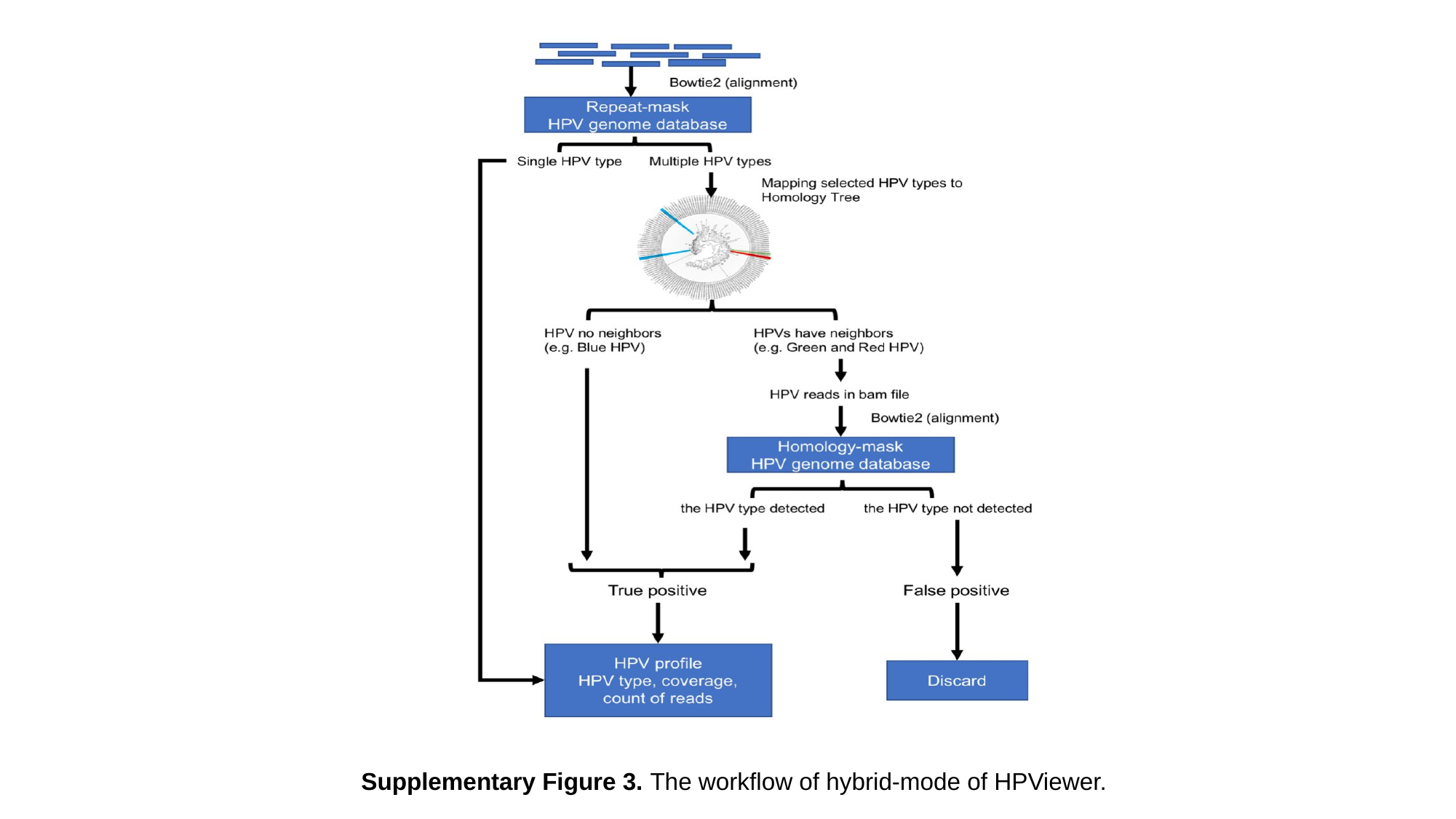

Supplementary Figure 3. The workflow of hybrid-mode of HPViewer.
